# Jejunal limb obstruction by a tumor thrombus from pancreatic metastasis of renal cell carcinoma: a case report

**DOI:** 10.1186/s40792-021-01122-w

**Published:** 2021-02-03

**Authors:** Shinya Sakamoto, Masaru Matsumura, Keigo Tani, Satoshi Nemoto, Kazuhito Tsuchida, Fumitaka Koga, Yasuji Seyama

**Affiliations:** 1 Department of Hepato-Biliary-Pancreatic Surgery, Tokyo Metropolitan Cancer and Infectious Diseases Center, Komagome Hospital, 3-18-22 Honkomagome, Bunkyo-ku, Tokyo, 113-8677 Japan; 2 Department of Gastric Surgery, Tokyo Metropolitan Cancer and Infectious Diseases Center, Komagome Hospital, Tokyo, Japan; 3 Department of Urology, Tokyo Metropolitan Cancer and Infectious Diseases Center, Komagome Hospital, Tokyo, Japan

**Keywords:** Pancreatic metastasis, Renal cell carcinoma, Palliative surgery, Pancreatectomy, Jejunal limb obstruction, Bowel occlusion

## Abstract

**Background:**

Renal cell carcinoma (RCC) is a primary tumor with the highest frequency of pancreatic metastasis. Although surgical resection can improve the prognosis of some patients with pancreatic metastasis of RCC (PM-RCC), the role of palliative surgery remains unclear. Herein, we described a case of jejunal limb occlusion caused by a tumor thrombus arising from a PM-RCC which was treated by surgical resection.

**Case presentation:**

A 75-year-old, male patient with metastatic RCC was admitted to our hospital with new-onset dysphagia and weight loss. Twenty years earlier he underwent a right nephrectomy with an adrenalectomy for the first surgical resection of RCC, and 12 years ago he underwent a left partial nephrectomy for metachronous primary RCC. Nine years later, multiple pancreatic metastases were detected. After discontinuing interferon therapy, he was followed up at his request without anticancer treatment. Multiple, pulmonary metastases developed 3 years ago, and resection of a brain metastasis was performed 6 months ago. He had also undergone a total gastrectomy with Roux-en Y reconstruction and splenectomy for gastric cancer 23 years ago. Computed tomography revealed a metastatic lesion in the pancreatic tail extending into the jejunal limb, which was obstructed by a tumor thrombus. Jejunal limb resection was performed concomitantly with a distal pancreatectomy as palliative surgery. The jejunal limb remnant was approximately 30 cm long and was re-anastomosed to the esophagus using a circular stapler. Blood perfusion at the anastomotic site was confirmed by indocyanine green fluorescence imaging. He was discharged on postoperative day 24 and was followed in the outpatient clinic. He achieved sufficient oral intake at 8 months postoperatively.

**Conclusions:**

PM-RCC can invade the gastrointestinal tract and cause tumor thrombus formation resulting in bowel occlusion requiring surgical intervention.

## Background

Renal cell carcinoma (RCC) is a primary tumor with the highest frequency of pancreatic metastasis [1]. Surgical resection can improve the prognosis of patients with pancreatic metastasis of RCC (PM-RCC), but the role of palliative surgery remains unclear [2, 3]. PM-RCC invasion of the digestive tract is unusual. Herein we described a case of jejunal limb obstruction caused by a tumor thrombus arising from PM-RCC which was treated with palliative surgical resection.

## Case presentation

A 75-year-old, male patient with metastatic RCC was admitted to our hospital with new-onset dysphagia and weight loss. His first surgical resection of RCC was a right nephrectomy with adrenalectomy which he underwent 20 years ago, and the second resection was a left partial nephrectomy for metachronous primary left RCC performed 12 years ago. Nine years later, computed tomography (CT) showed a 4-mm, early enhancing tumor in the pancreatic head and a 6-mm, early enhancing tumor in the pancreatic tail. PM-RCC was diagnosed. Total pancreatectomy was proposed, but he declined it. He also declined receiving tyrosine kinase inhibitors and interferon therapy was started. Although its adverse events were minimal, the patient discontinued the treatment on his own discretion. He declined any further anticancer drug therapy and was followed up every 3 months as an outpatient. The pancreatic metastases gradually increased in size and number, but were asymptomatic. Multiple pulmonary metastases also appeared 3 years ago, and dyslexia caused by a brain metastasis developed 9 months ago. The brain metastasis was resected 6 months ago. The patient also had a history of a total gastrectomy with Roux-en Y reconstruction and a splenectomy for gastric cancer 23 years earlier.

**Fig. 1:**
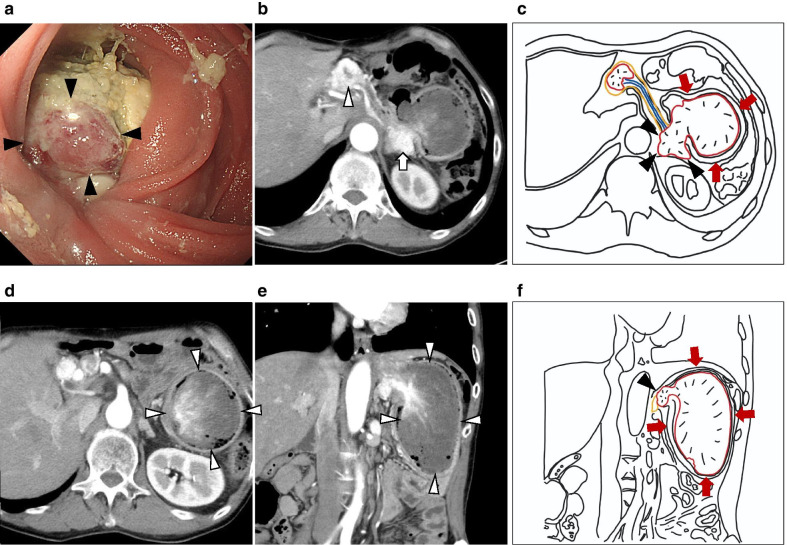
Esophagogastroduodenoscopy (EDGS) images and abdominal CT findings. **a** EDGS detected a massive tumor with necrotic tissue (arrowhead) in the jejunal limb occupying the lumen. **b**, **d**, **e** Images in the arterial phase of CT. **b** Hypervascular tumors can be seen in pancreatic head (arrowhead) and tail (arrow), and the massive tumor in the pancreatic tail can be seen extending into the jejunal limb. **c** A schema of **b**. The tumor in the pancreatic tail (arrowhead) invaded the wall of the jejunal limb and expanded into lumen of the jejunal limb (arrow). **d**, **e** The jejunal limb was enlarged and filled by the tumor thrombus (arrowhead). **f** A schema of **e**. The tumor in the pancreatic tail (arrowhead) invaded the wall of the jejunal limb and caused tumor thrombus filling the lumen of the jejunal limb (arrow)

His oral intake of food was insufficient, and his Eastern Cooperative Oncology Group performance status was 2. The laboratory tests on the day before the operation showed the patient had mild anemia (Hb 9.1 g/dL) and hypoalbuminemia (Alb 2.6 g/dL). His blood coagulation, kidney and liver function, and electrolytes were normal. Esophagogastroduodenoscopy demonstrated a massive, reddish tumor on the distal side of the anastomotic site in the lumen of the jejunal limb (Fig. 1a). CT revealed that one of the metastases in the pancreatic tail had markedly enlarged compared with previous CT. PM-RCC invaded the jejunal limb and extended into the lumen. A tumor thrombus completely filled the jejunal limb, increasing its diameter to 7 cm. The proximal side of the jejunal limb had also expanded, with the tumor thrombus causing bowel occlusion (Fig. 1b–f). Although preoperative biopsy of the tumor was not performed because of concern about hemorrhagic complications, based on CT findings and course of tumor growth, jejunal limb obstruction due to a tumor thrombus from PM-RCC was diagnosed. The size and number of the pulmonary metastases were stable, and no new metastases were detected.

Palliative surgery was considered as means of ameliorating the patient’s symptoms. However, because the tumor thrombus was located at a site immediately distal to the esophagojejunal anastomosis, bypass surgery would have been difficult. To remove the bowel occlusion, distal pancreatectomy with concomitant resection of the jejunal limb and re-anastomosis were considered to be necessary. Although the patient had multiple RCC metastases, his general condition remained fairly good, and tumor progression was slow. After the surgical indications were discussed in detail, palliative resection of the tumor thrombus was chosen as the method of treatment.

**Fig.2:**
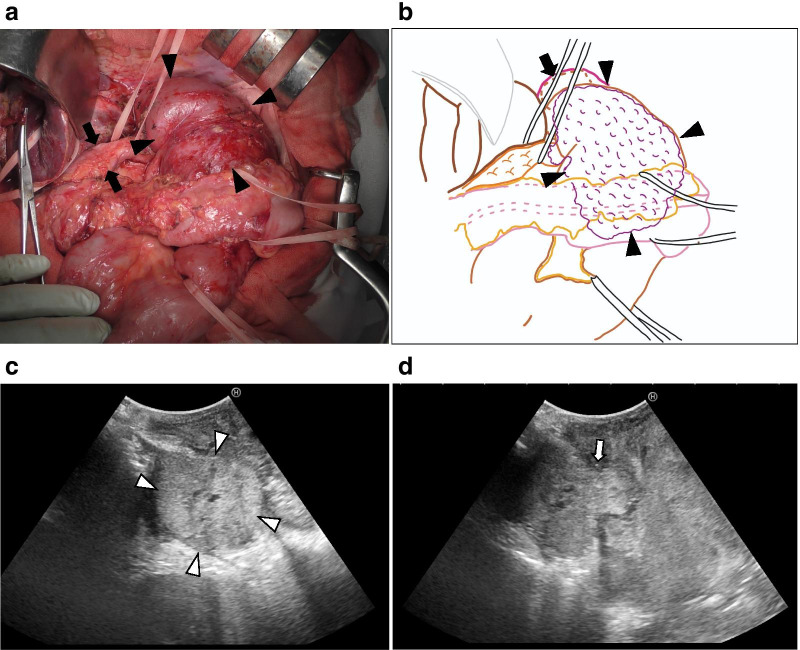
Intraoperative findings. **a** The jejunal limb (arrowhead) was enlarged and occupied by the tumor thrombus (arrow). **b** A schema of pancreatic metastasis. Pancreatic metastasis extended into the jejunal limb and caused a tumor thrombus filling the jejunal limb (arrowhead). The tumor thrombus was not reached at the abdominal esophagus (arrow). **c**, **d** Intraoperative ultrasound sonography. **c** Massive tumor in the pancreatic tail (arrowhead). **d** The tumor extended into the lumen of the jejunal limb (arrow)

**Fig. 3:**
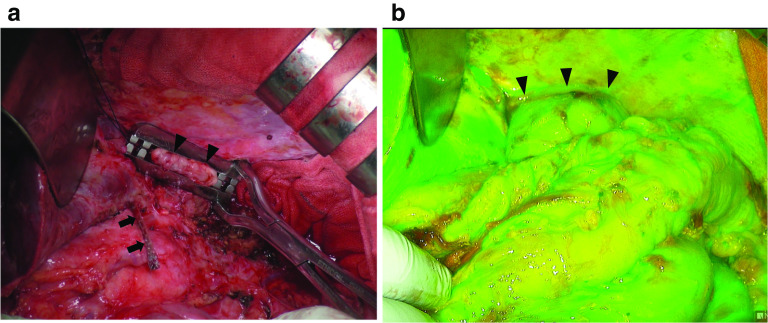
Intraoperative findings after resection and reconstruction. **a** After partial resection of the jejunal limb together with a distal pancreatectomy. The esophagus (arrowhead) was resected at the side proximal to the esophagojejuno-anastomosis. The pancreas was divided at the site immediately proximal to the tumor (arrow). **b** Indocyanine green (ICG) fluorescence imaging confirms sufficient blood perfusion of anastomotic site (arrow)

Exploratory abdominal surgery revealed no peritoneal metastases. First, adhesiolysis of the upper abdominal cavity was done. The jejunal limb showed enlargement due to the tumor thrombus, which extended from the pancreatic tail, and the jejunal wall showed thinning and pallor (Fig. 2a, b). Intraoperative sonography (IOUS) showed a massive pancreatic mass extending into the jejunal limb (Fig. 2c, d). To remove the patient’s symptoms, jejunal limb resection was performed concomitantly with a distal pancreatectomy. After dividing the pancreas using reinforced staples at the site closest to the tumor based on IOUS findings, the jejunal limb was resected from a point proximal to the jejunoesophageal anastomosis to the distal side of the tumor thrombus (Fig. 3a). The jejunal limb remnant was approximately 30 cm long and was re-anastomosed to the esophagus using circular staplers while avoiding excessive tension. Blood perfusion at the anastomotic site and transverse colon was confirmed by indocyanine green (ICG) fluorescence imaging (Fig. 3b).

**Fig. 4:**
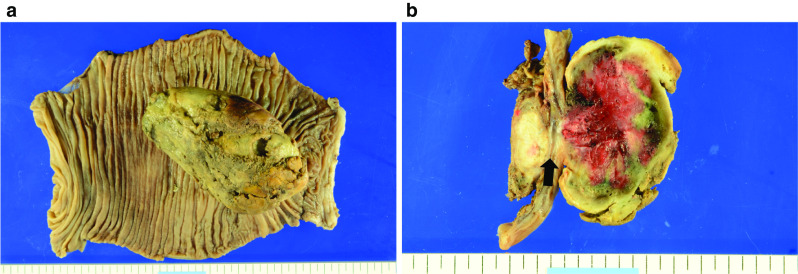
Resected specimen. **a** Surgical specimen from the distal pancreatectomy and jejunal limb resection. The tumor, 11.8 × 7.4 × 4.0 cm in size, was located in the jejunum. **b** Pancreatic metastasis of RCC had invaded the jejunal wall (arrow)

The patient’s postoperative course was uneventful except for an intraabdominal infection, which was treated with intravenous antibiotics. He was discharged after achieving sufficient oral intake on postoperative day 24. Histologic examination revealed metastatic RCC of the pancreas involving the jejunum (Fig. 4). At a follow-up examination in the outpatient clinic at postoperative 8 months, he had no gastrointestinal symptoms.

## Discussion

Renal cell carcinoma is one of the commonest tumors with pancreatic metastases and has an incidence of 0.8–3% in patients with a pancreatectomy [1, 4]. Several studies have reported that the presence of a pancreatic metastasis is not a risk factor of poor prognosis in RCC [5, 6]. Regardless of the feature with systematic disease, its long-term outcome is not very poor, as seen in the 5-year overall survival rate of 78.9% in patients undergoing a nephrectomy following the diagnosis of a pancreatic metastasis [7]. However, the prognosis of patients with concomitant, multiple organ metastases is generally dismal [8]. In the present case, the PM-RCC invaded the jejunal limb after Roux-en Y reconstruction and formed a tumor thrombus causing bowel occlusion. Palliative surgery was performed, and the patient experienced a generally favorable postoperative course.

There are few reports of PM-RCC invading the digestive tract [10, 11]. Gajendra et al. reported a case of PM-RCC in the uncinate process which invaded the duodenum and caused gastrointestinal bleeding [9]. George et al. described a case of PM-RCC in the uncinate process which invaded the third portion of the duodenum, causing small bowel occlusion [10]. PM-RCC has the potential to invade the adjacent gastrointestinal tract directly. In the present case, once the PM-RCC had invaded the adjacent jejunal limb, it gradually expanded, forming a tumor thrombus that caused bowel occlusion. One of the unique features of RCC is the formation of a tumor thrombus which can migrate into the venous system [11]. To the best of our knowledge, the present study is the first to report bowel occlusion caused by a tumor thrombus arising from PM-RCC. The unusual progression of the disease in the present case might be explained by the tumor’s expansive growth in the lumen, which is characteristic of RCC.

The Japanese guidelines recommend that a metastasectomy be restricted to patients who can be expected to achieve complete tumor removal [12], and the role of palliative surgery in the context of symptomatic PM-RCC remains unclear [13]. Palliative surgery might provide relief from the symptoms of bowel occlusion and enable resumption of oral food intake [14]. The present patient had multiple, unresectable, distant metastases although the disease did not rapidly progress for many years despite the lack of systemic anticancer therapy. The patient was therefore expected to survive longer than 6 months, leading to the decision to perform palliative surgery. Since his operation, the patient has maintained adequate oral food intake for more than 6 months.

In general, palliative surgery for gastrointestinal occlusion includes bypass surgery or resection and re-anastomosis. A previous report demonstrated that a gastrojejunal bypass can be effective for treating a duodenal obstruction due to direct, PM-RCC invasion [10]. However, in the present case, the patient had a history of gastrectomy, and the wall of his jejunal limb showed thinning and pallor in the area immediately distal to the esophagojejunal anastomosis due to the massive tumor thrombus in the lumen. A jejunojejunal bypass was therefore impossible. After meticulous adhesiolysis around the portion occupied by the tumor thrombus, the jejunal limb both was able to be encircled at the oral and anal sides of the obstruction and resected together with the distal pancreas.

Appropriate tension and sufficient blood supply at the anastomotic site are essential for proper healing [15]. In the present case, the length of the jejunal limb after resection, including the anastomosis, was adequate to re-anastomose the jejunum to the esophagus without excessive tension. ICG fluorescence imaging, which is used to asses gastrointestinal anastomotic blood perfusion [16] and anastomotic blood flow in patients with a gastrectomy [17], demonstrated adequate blood perfusion at the anastomosis site in the present patient.

## Conclusions

The present report described a case of bowel occlusion caused by a tumor thrombus stemming from PM-RCC. A pancreatic metastasis of RCC has the potential to invade the gastrointestinal tract and give rise to a tumor thrombus resulting in bowel occlusion requiring surgical intervention.

## Abbreviations

RCC: Renal cell carcinoma
PM-RCC: Pancreatic metastasis of renal cell carcinoma
CT: Computed tomography
IOUS: Intraoperative sonography
ICG: Indocyanine green
